# Avocado toast and pot roast: exploring perceptions of generational communication differences among health sciences librarians

**DOI:** 10.5195/jmla.2020.851

**Published:** 2020-10-01

**Authors:** Rachel Keiko Stark, Jenessa McElfresh

**Affiliations:** 1 stark@csus.edu, Health Sciences Librarian, University Library, California State University, Sacramento, Sacramento, CA; 2 jmcelfr@clemson.edu, Health Sciences Librarian, Clemson University Libraries, Clemson University, Clemson, SC

## Abstract

**Objective:**

This research explored health sciences librarians' perceptions of intergenerational communication in the workplace.

**Methods:**

The authors developed and sent a survey to health sciences librarians through email discussion lists from August 2018 to September 2018. A chi-square test was performed to determine whether one's length of employment as a librarian was associated with belief that age and/or generation impacts communication in the workplace.

**Results:**

A total of 150 respondents from 5 countries completed the survey. There was no significant association between length of employment as a librarian and respondents' belief that age and/or generation impacts communication in the workplace. However, regardless of length of employment, most respondents indicated that generational differences do have an impact on communication in the workplace. Also, most respondents expressed interest in institutional initiatives to foster intergenerational communication.

**Conclusion:**

The authors found that health sciences librarians believe that differences among generations impact communication in the workplace. Librarians, managers, and library organizations should consider providing training and other opportunities for health sciences librarians to improve their intergenerational communication skills.

## INTRODUCTION

Communication between employees is a central component of ensuring a functional, productive workplace. In libraries, navigating internal conflict, managerial pressure, and external pressures can have a direct impact on employee retention, productivity, and user comfort in utilizing library resources [1]. For health sciences or medical information professionals, this impact is acutely felt due to the nature of these jobs requiring internal (library) communication in addition to frequent interaction with external (hospital administration, academic hierarchies) sources of support and collaboration. While general communications skills have long been deemed to be an essential area of expertise in librarianship and have much support in continuing education and training, perceptions of the skills and training needed to explore communications between generations has been greatly overlooked [2].

Intergenerational communication is defined broadly as interactions between individuals of differing generations. According to the *SAGE Encyclopedia of Communication Research Methods,* the impact of studies on intergenerational communication has led to “understanding of antecedents, motivations, processes, and consequences of communication across generations, and the ways in which individual characteristics and/or social/historical context jointly shape our interpretations of and responses to such interactions” [3]. This has profound implications in libraries, including in the unique workplace interactions and assistance of patron groups that utilize health sciences libraries. In the health sciences library context, intergenerational communication can take the form of interactions between direct coworkers, supervisors, subordinates, library users, and external governing bodies and collaborators.

The authors' previous literature review found no research investigating intergenerational communication in health sciences librarians' work experiences [4]. This study sought to fill this gap by serving as an initial, broad exploration into the perceptions and experiences of health sciences librarians related to generational labels and intergenerational communication in the workplace.

Our hypothesis was that length of employment as a health sciences librarian at a given institution influences perception of the impact of age and/or generational communication in the workplace. This hypothesis was formed based upon qualitative data collected at an interactive poster session, in which we directly engaged with health sciences librarians and received feedback via verbal and written responses about librarians' experiences with intergenerational communication in the health sciences workplace [5]. Beyond this hypothesis, our study results revealed the presence of broader interest in and perceived conflicts regarding intergenerational interactions in health sciences libraries, demonstrating the ongoing necessity of inquiry into intergenerational studies in the library workplace.

## METHODS

Our study was conducted via an electronic survey on the Qualtrics platform. The survey had twenty-one questions; seventeen questions had Likert scale responses; and four questions had free-text responses (supplemental appendix). We used the term “health sciences library” or “health sciences librarian” to refer to any library or information professional with a focus on health care, medicine, or related allied health disciplines in their professional settings.

Though generations can be defined based on age groupings, relationships, or developmental stages, this study primarily addressed generations by age groupings, as determined by year of birth and defined by the Pew Research Center [3, 6]. The Pew Research Center is a nonpartisan, nonprofit organization and, thus, provides less biased, freely available generational definitions. As the Pew Research Center generational data are freely available online to the general public, respondents could choose to verify the definitions that we provided, as the precise years associated with generational labels sometimes change, based on the evolution of life experiences per generational label, or differ, based on reporting agency [6].

Two survey questions used the phrase “age and/or generation” to solicit respondents' perceptions of the influence of these factors in the workplace. For these questions, age and generation were conflated into one category, based on the Pew determinants of generation by age, and our desire to solicit the opinions of respondents who did not believe in generational labels, as first observed during the interactive poster session on the topic.

Areas of particular interest included possible influence of years in the profession on perception of the communication abilities of other librarians, possible impact of self-perceived age on communication with other librarians from different age groups, and self-perception of communication ability across generations. The survey included options for respondents to write in comments to collect limited feedback to enrich the authors' understanding of the collected data.

The survey was tested for ease of use and logical reliability by having librarians outside of the health sciences complete the survey, and the survey was then adjusted based on their feedback. The survey was submitted as part of the institutional review board (IRB) process at two different universities: Sacramento State University and Clemson University. After IRB approval of the project at both institutions, the survey was embedded in an email that included informed consent and our contact information. The email was sent out in August 2018 to the MEDLIB-L email discussion list and MLA chapter email discussion lists, such as the list of the Northern California and Nevada Medical Library Group and Southern Chapter of the Medical Library Association. Unexpectedly, the survey also became of interest to librarians in Canada, the United Kingdom, and Australia, with members of medical library groups from those countries contacting us and asking to send the survey to email discussion lists in their countries, to which we granted permission. The survey was closed after five weeks.

Survey data were analyzed using SPSS version 25, with statistical significance defined as a 2-tailed *p*<0.05.

## RESULTS

A total of 150 respondents completed the survey, with 28 additional incomplete responses, resulting in a completion rate of 84%. Due to the unknown number of email discussion lists to which the survey was sent through respondents forwarding it, the overall response rate was unknown. Most respondents were between 45–64 years old (51%), followed by 35–44 years (22%), 25–34 years (17%), 65 years or older (9%), and 24 years or younger (1%). Most respondents had more than 11 years of experience in health sciences libraries (56%). Librarians with more years of experience tended to be older. Nearly half (45%) of respondents worked in academic health sciences libraries, 36% worked in hospital libraries, and the others worked in general academic, corporate, or other settings. Reflecting the demographics of the library profession [7], most respondents identified as female (88%), almost 10% identified as male, and 1 respondent identified as nonbinary.

Most of the 150 completed responses were from librarians located in the United States; however, about 10% of completed responses were from countries around the world. There were 5 responses from Canada, 5 from Australia, 4 from New Zealand, and 2 from the United Kingdom.

**Figure 1:**
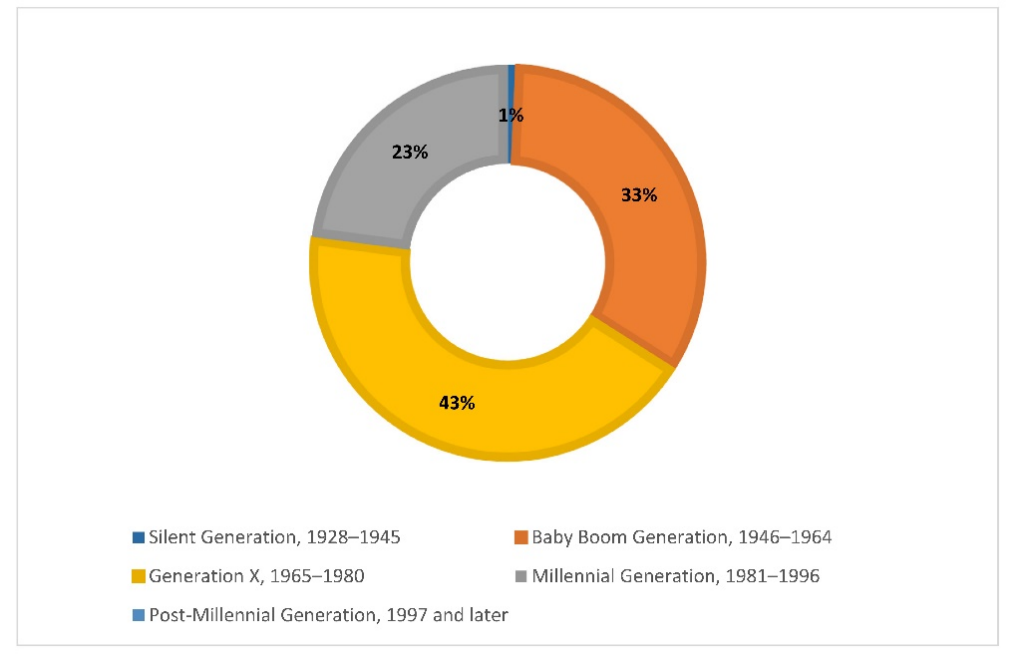
Survey respondents' self-identified generational identity

The survey asked respondents to identify the generation with which they most closely identified. Most respondents (43%) identified as Generation X, 33% identified as Baby Boomers, 23% identified as Millennials, and 1% identified as being a member of the Silent Generation. No respondents identified as a member of the Post-Millennial generation (Figure 1).

In free-text responses, respondents reported a variety of opinions regarding their agreement with their generational labels. Some self-identified with the generational label assigned to their year of birth, for example, “I am the quintessential baby boomer. My Dad served in WWII.” Others agreed with the label but disagreed with its accompanying stereotypes, for example, “I do feel that I am a millennial, but I do not feel as though I fit the ‘stereotypes' of the millennial generation.” Other responses seemed somewhat contradictory, such as “Generations are a social construct that needlessly separate our society but at least most millennials aren't hateful about it like people in other age groups I've seen in general.” Not all respondents identified with their generational labels and provided commentary such as “I've never felt like I fit into the Gen X characteristics.”

**Figure 2:**
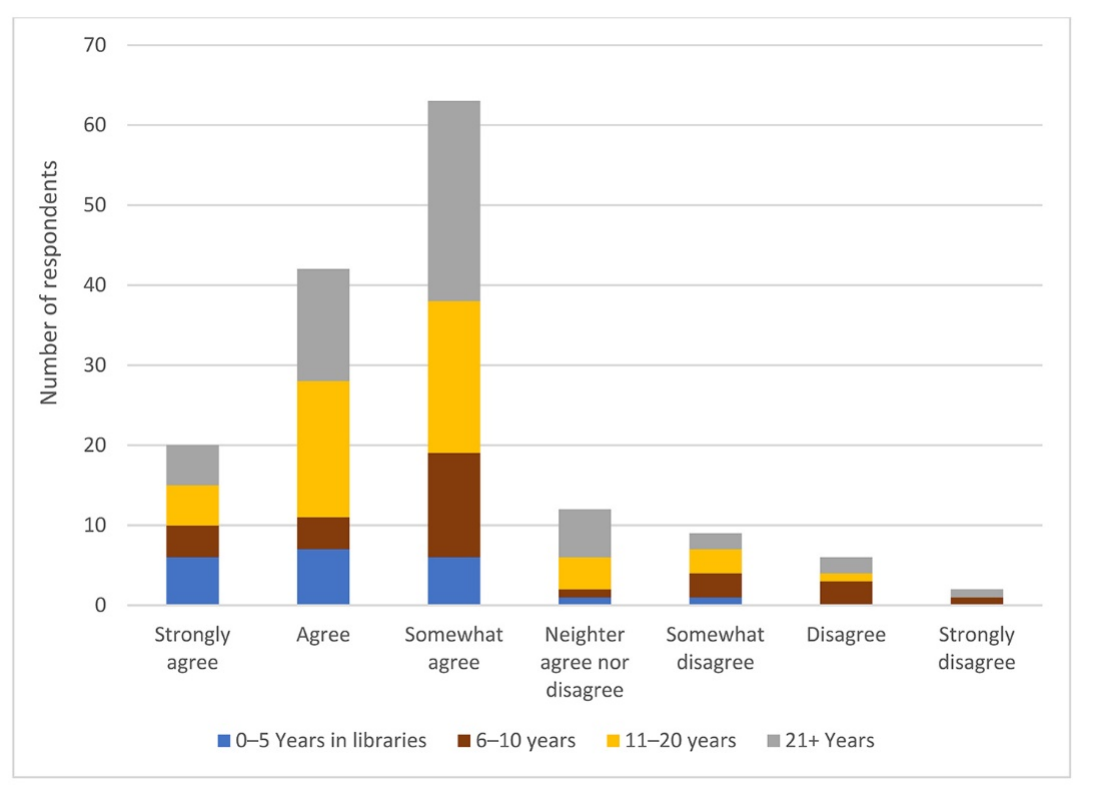
Respondents' belief that age and/or generation has an impact on communication in the workplace based on years worked in a library

**Figure 3:**
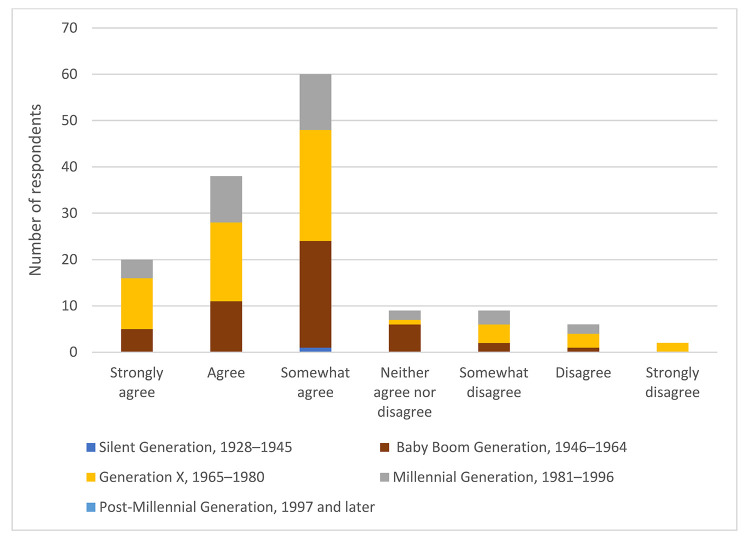
Respondents' belief that age and/or generation has an impact on communication in the workplace based on respondents' generation

**Figure 4:**
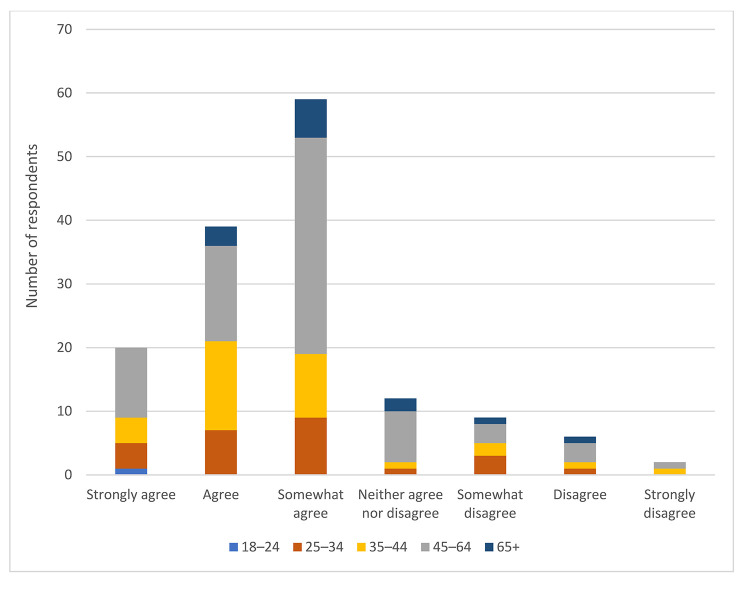
Respondents' belief that age and/or generation has an impact on communication in the workplace based on respondent age

Overall, 13% of respondents strongly agreed, 28% agreed, and 25% somewhat agreed that age and/or generational differences have an impact on communication in the workplace. However, we found no significant relationship between one's length of time in the library profession and belief that age and/or generational differences had an impact on communication in the workplace (Χ^2^(18)=0.396, *p*>0.05) (Figure 2). Similarly, there were no significant relationships between one's generation (Figure 3) or age (Figure 4) and belief that age and/or generational differences have an impact on communication in the workplace.

**Figure 5:**
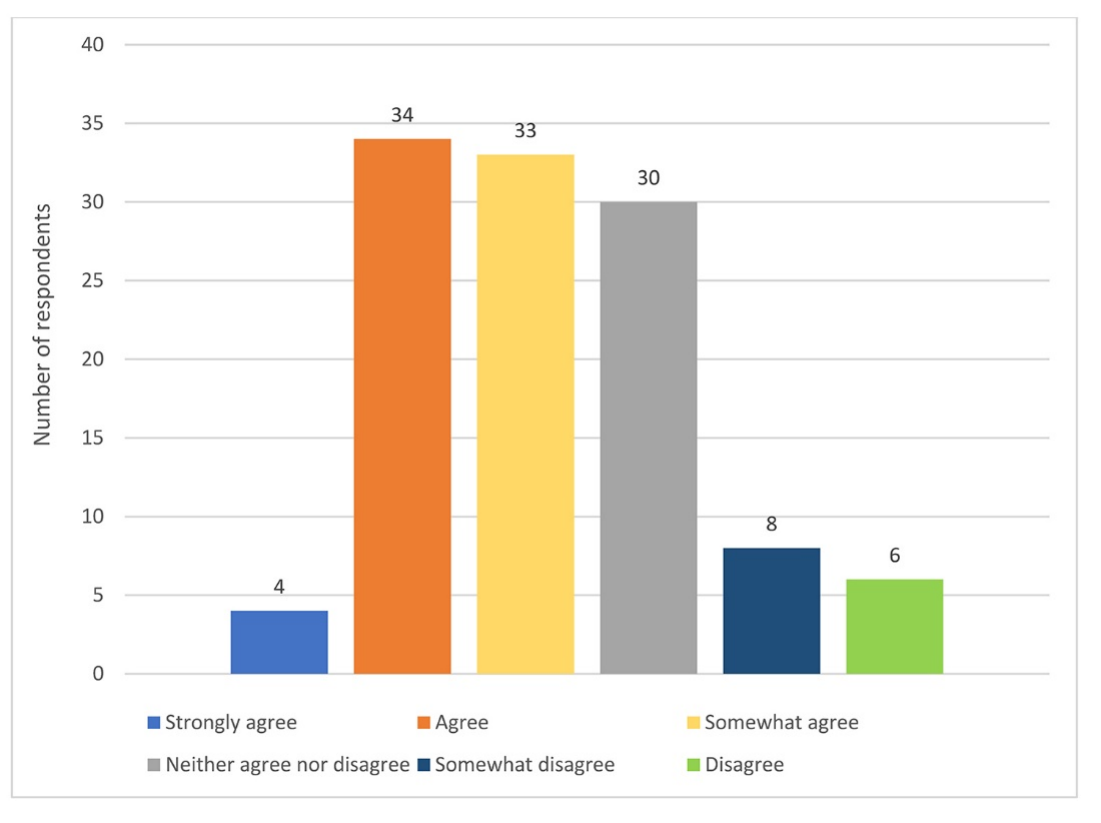
Respondents' interest in institutional initiatives to foster intergenerational communication

Although most respondents believed that age and/or generation had an impact on communication in the workplace, a minority of respondents (22%) reported that their institutions or libraries took actions to foster communication between people of different generations. Of those who responded affirmatively, the examples that they provided including trainings, voluntary institutional groups, low-stakes social settings, programs offered through collaborations with diversity and inclusion groups, mentoring scenarios, and social settings in which intergenerational collaboration was an outcome but not a stated goal. Also, most respondents (60%) strongly agreed, agreed, or somewhat agreed that they would find institutional initiatives to foster intergenerational communication helpful (Figure 5).

## DISCUSSION

We found no statistically significant association between a health sciences librarian's length of employment and their perceptions of how age and/or generation communication issues impacted the workplace experience. Although length of employment in a library did not impact these generational perceptions, the survey results suggested that facets of age and generation impacted how health sciences librarians perceived their interactions with colleagues, regardless of their length of employment. Thus, the collected information offered interesting insights and provided opportunities for reflection regarding communication among generations in the health sciences library workforce.

The varying differences in opinion on generations in the health sciences library workplace that were expressed by survey respondents in open ended responses indicated that there was not a cohesive attitude toward generations in the health library workforce. The varied and at times opposing opinions on intergenerational communication in libraries was revelatory in that it highlighted the inherent conflict in exploring the topic and the necessity of continued workplace training and conversations to make sure that all viewpoints are validated and treated with respect.

Although underexplored among health sciences librarians, interactions between generations have been explored in other professions as an extension of communications studies and the unprecedented age diversity in the twenty-first century workforce [8]. For example, among nurses, generational divides have been reinforced through socialization and on-the-job training [9]. In both the United States and Thailand, bankers who were older employed more negative and commanding communication [10]. Furthermore, different generations of hospitality workers in North America have shown different preferences for types of communication in the workplace [11]. This research in other professions echoed the themes that were explored in this study, exploring the impact of generational identity on workplace communication and other aspects of professional conduct. As related studies in other fields attest, intergenerational communication was perceived by respondents to have a demonstrated impact on the working environment of health sciences librarians [12].

In the present study, respondents were asked to specify their generational label based on the years assigned by the Pew Research Center [6] and were then asked if they agreed with this label and given space to elaborate. We understand that this topic can be contentious and wanted to ensure that respondents had the opportunity to provide clarifying information. The open-ended responses suggested that although most respondents agreed with their Pew-defined generational label, a substantial portion of respondents either did not agree or had mixed feelings regarding generational labels in general.

Definitions of generational labels (e.g., “Baby Boomer,” “Generation X”) beyond their year ranges were not provided in the survey, yet respondents freely used generational labels in their open-ended responses, indicating their familiarity with these labels. In the open-ended responses, we found widespread stereotyping and generalizations associated with different generations, reflecting the pervasiveness of generational typecasting. Respect, empathy, and social or personal responsibility were universal traits that respondents noted as superseding that of generational labeling, demonstrating the perception of generational labels as being inflexible constructs, rather than being inclusive of ethics, morality, and personal experiences.

A limitation of our study was the wide variety of staffing models used in health sciences and medical libraries, which made it difficult to interpret the quantitative data on intergenerational interactions in the workplace. In retrospect, we would have liked to ask respondents about their workplace models (e.g., solo librarian, team-based) to provide clarification. Another limitation was the lack of a validated instrument to measure intergenerational communication in the library workplace. Due to the unique work of health sciences librarians—and hospital librarians, in particular—we felt that the use of an instrument validated in a different profession would not accurately capture the experiences of health sciences librarians. Thus, development of a validated instrument for health sciences librarians could benefit future research.

Our experience performing this survey and its results indicate that intergenerational communication is a topic that health sciences librarians are eager to explore, indicating the need for further research, outreach, and training. Future research could assess the impact of trainings and interventions on health sciences librarians' skills and comfort level in intergenerational communication in the workplace.

## SUPPLEMENTAL FILE

**Appendix:** Survey
