# Supplementary material for: Avocado toast and pot roast: exploring perceptions of generational communication differences among health sciences librarians
Source: J Med Libr Assoc. 2020 Oct 1;108(4):591–7. doi: 10.5195/jmla.2020.851 (PMC7524637; doi:10.5195/jmla.2020.851)
Supplement: Supplementary file 1 — Appendix: Survey [file jmla-108-4-591-s01.pdf]

JMLA Journal of the Medical Library Association

SC
Supplemental content to
J Med Libr Assoc. Oct;108(4):dx.doi.org/10.5195/jmla.2020.851
www.jmla.mlanet.org
© Stark, McElfresh 2020

Avocado toast and pot roast: exploring perceptions of generational communication differences among health sciences librarians

Rachel Stark, AHIP; Jenessa McElfresh, AHIP

APPENDIX

Survey

Questions

1. How long have you worked in libraries?
   0–5 years 6–10 years 11–20 years 20+ years
   Are you retired?
   Y or N

2. In what city/state is your workplace located? ______

3. How long have you been employed at your current institution or most recent institution?
   0–1 years 2–5 years 6–10 years 11–15 years 16–20 years 21+ years

4. What type of library do you work at?
   Hospital Public Health sciences academic General academic Public health Pharmacy
   Corporate Other ______

5. What is your job title? ______

6. How many people at your institution have a job title similar to yours?
   0 1–3 4–9 10+

7. How many part-time and full-time employees work in the library where you are currently employed? ___

8. Do you think the people you work with on a daily basis at your institution represent a wide range of ages?
   a. Yes
   b. No

9. Have you encountered a difficult situation in the past six months communicating with a colleague who is:
   a. older than you?
   b. younger than you?
   c. same age as you?
   d. If so, please tell us more ______

J Med Libr Assoc. Oct;108(4):dx.doi.org/10.5195/jmla.2020.851
www.jmla.mlanet.org
© Stark, McElfresh 2020

10. Do you supervise people, either employees or volunteers?
    a. Do you feel you have experienced difficulties communicating with the people you supervise due to generational differences? ______

11. If you work directly with a supervisor, in your estimation, is your supervisor older or younger than you?

______

12. Have you encountered communication difficulties with someone who shares a similar job title to you?
    a. If yes,
        i. Do you think that generational differences contributed to the difficulties? ______

13. Does your library or institution do anything to foster communication between people of different generations?
    a. If yes,
        i. Please elaborate ______
        ii. Have you found it effective? ______
            1. Definitely yes Definitely not

14. Would you find institutional initiatives to foster intergenerational communication helpful? ______

15. Do you believe that age and/or generation has an impact on communication in the workplace?
    a. Strongly agree Strongly disagree

16. Do you believe that age and/or generation has an impact on the ability of librarians to provide quality services to library users?
    a. Yes
    b. No
    c. If you would like to elaborate, please do so ______

Demographic questions

17. What is your age?
    18–24 25–34 35–44 45–54 55–64 64+

18. Please select the gender you most identify with:
    a. Male
    b. Female
    c. Nonbinary
    d. Other

JMLA Journal of the Medical Library Association

SC
Supplemental content to
J Med Libr Assoc. Oct;108(4):dx.doi.org/10.5195/jmla.2020.851
www.jmla.mlanet.org
© Stark, McElfresh 2020

19. What geographical space most impacts your identity?
    a. My country: ______________________________
    b. My state/province: ______________________________
    c. My city: ______________________________

20. What generation do you most identify with? (Please circle)

* In actual survey, chronological order, oldest first.

The generations defined

The Millennial generation
Born: 1981 to 1998
Age of adults in 2016: 18 to 35*

Generation X
Born: 1965 to 1980
Age in 2016: 36 to 51

The Baby Boom generation
Born: 1946 to 1964
Age in 2016: 52 to 70

The Silent Generation
Born: 1928 to 1945
Age in 2016: 71 to 88

The Greatest Generation
Born: 1901 to 1927
Age in 2016: 89 to 115

* The youngest Millennials are in their teens. No chronological end point has been set for this group

PEW RESEARCH CENTER

21. Do you agree with the generational label assigned to your year of birth?
    a. Yes
    b. No
    c. If no, please elaborate ______________________________
